# When Bones Blur the Lines: Ancient DNA Validation of Morphological Sex Estimation Traits and the Challenges of Population-Specific Dimorphism

**DOI:** 10.3390/genes17070726

**Published:** 2026-06-23

**Authors:** Francisca Alves-Cardoso, Cláudia Gomes, Sara Palomo-Díez, César López-Matayoshi, Steffi Vassallo, Anne Malcherek, Zélia Rodrigues, Sandra Assis, Nicholas Márquez-Grant

**Affiliations:** 1 Laboratory of Biological Anthropology and Human Osteology (LABOH), Centro em Rede de Investigação em Antropologia (CRIA)/IN2PAST, Polo NOVA FCSH/School of Social Sciences and Humanities, Universidade Nova de Lisboa, Av. de Berna, 26-C, 1069-061 Lisboa, Portugal; svassallo@campus.fcsh.unl.pt (S.V.); annemalcherek@campus.fcsh.unl.pt (A.M.); sassis@fcsh.unl.pt (S.A.); 2 Legal Medicine, Psychiatry and Pathology Department, Medicine School, Complutense University of Madrid (UCM), 28040 Madrid, Spain; spalomod@ucm.es (S.P.-D.); clopezma@ucm.es (C.L.-M.); 3 Department of Cellular and Molecular Sciences, Faculty of Sciences, Universidad Peruana Cayetano Heredia, Lima 15102, Peru; 4 3D Printing Center for Health, School of Science and Technology, NOVA University Lisbon, 2829-516 Caparica, Portugal; 5 Departamento Municipal de Gestão do Património Cultural (C. M. Porto), 4049-001 Porto, Portugal; 6 Arqueologia e Património Lda, 4455-804 Matosinhos, Portugal; 7 Egas Moniz School of Health and Science, Instituto Superior de Ciências da Saúde Egas Moniz, 2829-511 Monte da Caparica, Portugal; 8 School of Anthropology and Museum Ethnography, University of Oxford, Oxford OX2 6PE, UK; n.marquezgrant@arch.oxon.net

**Keywords:** sexual dimorphism, bioarchaeology, sex estimation, human variation, human remains, Porto (Portugal), aDNA

## Abstract

Background/Objectives: Sex estimation is a cornerstone of research and practice in bioarchaeology and forensic anthropology. However, morphological and metric methods are often hampered by population-specific variation, subjectivity in assessment, and taphonomy. This study compares morphological analysis and ancient DNA (aDNA)-based sex assessment in a 19th-century Portuguese sample to evaluate the accuracy of osteological (anthropological) criteria. Methods: This study analysed 37 skeletons from the Venerável Ordem Terceira da Nossa Senhora do Carmo burial grounds in Porto. Sex estimation was based on (1) the bioanthropological assessment of morphological traits of the os coxae and the skull (2) through aDNA analysis using a multi-marker approach, including real-time PCR (qPCR) targeting autosomal loci, the amelogenin locus, a Y-chromosomal INDEL, and Y-STRs. aDNA was extracted via a non-destructive protocol. Results: Whilst anthropological analysis was possible on all 37 individuals, estimation of sex through aDNA analysis was possible for 26 individuals. A 20% discordance rate was found between morphological and aDNA results. Many individuals morphologically classified as “possible female” or “indeterminate” were genetically identified as male. Genetic analysis resolved most cases that biological anthropologists concluded were “indeterminate”. Conclusions: The high discordance in the Carmo sub-sample may indicate reduced skeletal sexual dimorphism, with males exhibiting skeletal traits typically associated with females, suggesting a sample-specific reduction in sexual dimorphism likely influenced by environmental, nutritional, and/or genetic stressors. A limitation of this study is its small sample size: only 26 of 37 individuals yielded usable genetic results, and only a portion of these individuals provided sufficient data for a direct comparison between morphological and genetic data. Nevertheless, these findings highlight the risk that applying generalised osteological standards relying solely on morphology can lead to systematic misclassification, emphasising the need for a critical, multidisciplinary approach to sex estimation.

## 1. Introduction

Sex estimation is fundamental for understanding mortality, morbidity, and cemetery organisation, in addition to other questions in anthropology and bioarchaeology, and for the identification of the deceased in forensic anthropology [1,2,3,4,5,6] and genetics [7,8,9,10,11]. In biological anthropology, the majority of methods for sex estimation rely on the qualitative and quantitative morphological assessment of osteological traits [5,12,13,14,15,16]. The use of such morphological traits is essential in contexts where human remains are recovered in incomplete, fragmentary, or in a poor condition, often lacking the pelvic elements required for high accuracy, or when faced with commingled human remains. In such cases, alternative skeletal elements, such as long bones, are used; while dimorphic, the methods developed on these skeletal elements generally provide lower accuracy and are more sensitive to environmental factors and lifestyle. However, recent approaches relying on machine learning are securing high accuracy rates [17,18,19,20,21,22]. Since the standard osteological criteria for sex estimation are often based on specific reference populations, such as 20th-century North American or European collections [23,24,25,26,27], the reliability of these methods is compromised by population-specific variation, growth impairments, pathological conditions, and the full understanding of the factors that may influence the expression of traits used for sex estimation purposes. Most important is the fact that the application of these universal standards (i.e., selected sex-specific morphological and/or anatomical features) to diverse archaeological and historical populations can lead to systematic misclassification if the target population exhibits different patterns of sexual dimorphism: the key is to acknowledge population variability as an important factor in anatomical feature expression [28,29,30]. Furthermore, trait scoring is subjective and based on opinion, often tied to a person’s experience and understanding of trait expression, or to a personal bias about what constitutes a gracile or robust trait [31]. Biological ageing further complicates skeletal sex estimation. For example, due to post-menopausal hormonal changes, older women may present skulls with more robust features that are traditionally classified as being more “masculine” in nature. Phillip Walker argued that this diagnostic bias often reflects deep-seated ‘sexism in sexing’, suggesting that errors frequently stem from rigid cultural stereotypes of female morphology rather than nuanced understanding of the biological complexities inherent in human cranial dimorphism [32]. The emphasis on robusticity is a bias that often leads to the misidentification of large robust remains as male and small gracile remains as female, a dichotomy rooted more in Western cultural perspectives than in biological certainty. In reality, sexual dimorphism and robusticity are not synonymous [33]. Furthermore, skeletal robusticity is frequently a byproduct of biomechanical stress and the hypertrophy of musculoskeletal attachment sites, and/or disease-related bone changes [34,35,36,37,38]. Although genetic analysis has provided accurate means of sex estimation in recent years, which has allowed for the validation and refinement of osteological-based methods [7,39,40,41,42], some scholars have highlighted limitations such as the state of preservation of the remains [28,43,44]. Also important has been the role of bioanthropological research in resisting the theoretical framework that reduces people to isolated anatomical or chromosomal variables, by shifting from “sex determination” to “sex estimation”. [45]. This change frames genetic analysis as an interpretive scientific practice rather than the passive uncovering of an absolute biological mandate, allowing researchers to contextualise molecular data within complex genetic research and lived human realities. Ultimately, this bioanthropological framework ensures that genetic data does not reduce historical identity to a chromosomal binary and ancient DNA (aDNA) “objectification” of people, but that it is instead used responsibly to enrich our understanding of the multi-dimensional lives of past populations.

This current paper aims to contribute to the broader discussion of population-specific variation and the implications of sex-assessment biases for research in bioarchaeology and also in forensic anthropology. Developed within the scope of the BeFRAIL research project [46], although it was not the primary focus of the research, this study assists in the development of sample-specific sex estimation methods, whilst evaluating whether widely used sex estimation methods developed in the 20th century are applicable for analysing human remains from the Venerável Ordem Terceira da Nossa Senhora do Carmo cemetery in Porto (henceforth, the Carmo burial site). This study addresses the genetic and specifically aDNA validation of morphological sex estimation traits used in anthropology on a 19th-century Portuguese sample of human remains from the abovementioned burial site. The population from this period experienced significant physiological, environmental, and nutritional stressors, largely due to war and epidemics [47,48,49], which are known to impact skeletal development and the expression of skeletal morphological traits [50,51].

## 2. Methodological Approach

The individuals selected for this study were excavated from the Carmo burial site. Around 1047 primary burials were excavated as a result of development, with 429 individuals recovered during the first excavation that took place between 2006 and 2008 [52], and 618 during the second excavation (2022 to present). Alongside the primary burials, many remains were found in commingled contexts [53]. The remains are currently under analysis and therefore no definite results may be presented for the entirety of the sample. This study is based on a sub-sample of 37 individuals from primary burials. Twenty-four individuals were excavated during the first excavation and the remaining thirteen were recovered during the ongoing archaeological excavation. This sub-sample was selected randomly, and an evaluation of aDNA preservation potential was prioritised, hence both adults and non-adult individuals were included.

### 2.1. Bioanthropological Sex Estimation

The sub-sample selected for this study included 34 adults, defined by complete skeletal maturation, including full epiphyseal fusion and complete dental development, and 3 non-adults exhibiting incomplete dental and skeletal maturation. Skeletal development was assessed using standard anthropological methods outlined by Buikstra and Ubelaker (1994), Cunningham and colleagues (2016), and Schaefer and colleagues (2009) [1,54,55]. When referring to biological skeletal/bone development, we utilise the biological states mature and immature, and when expressing a demographic classification based on that development, we use the term adult and non-adult.

Skeletal sex estimation was performed via macroscopic assessment of the os coxae, cranium, and mandible morphological traits. Due to differential preservation across individuals and specific anatomical regions of interest (the os coxae and skull), a combination of several widely accepted and validated bioanthropological methods were used [1,6,13,56]. The osteological assessment followed standard protocols as per the cited authors, with a primary focus on the morpho-functional traits of the sacro-pelvic, acetabular, and ischio-pubic regions of the os coxae. Since the os coxae are credited as being highly sexually dimorphic [5,13,57,58], particular attention was given to the greater sciatic notch (shape and proportions), the composite arch, ischio-pubic ramus proportions, pubic shape, ventral arc, subpubic angle, subpubic concavity, the shape of the obturator foramen, and ischial and acetabular morphology [5,13,57,58]. This analysis was complemented with the evaluation of additional morphological features of the cranium and mandible, including the overall size and architecture, frontal and parietal eminences, forehead expression and robusticity of the supraorbital ridge/glabella, orbits—shape and thickness of the supraorbital margin, nasal aperture—size and shape, mastoid process—size and shape, zygomatic extension, nuchal crest, mental eminence, mandibular angle and gonial eversion [1,6]. Overall classification was of individuals being female, possible female, indeterminate, possible male, or male. As the most discriminating element of the skeleton, sex estimation was predominantly performed based on the morphology of the os coxae. The skull was used as a complement for diagnosis [16,59].

### 2.2. Genetic Sex Estimation

Two bone or tooth samples were selected from each individual to allow for duplicate analysis and verification of the results. Preference was given to complete teeth free of cracks, fractures, or decay. These teeth were processed in their entirety, with every effort made to preserve their integrity during analysis. When teeth were unavailable, bone fragments, preferably from the petrous portion of the temporal bone or a long bone diaphysis, were used. Bone fragments exceeding 2.5 cm on any side were sectioned down to facilitate processing.

#### 2.2.1. Sample Preparation and aDNA Extraction

Before aDNA extraction, all samples underwent a rigorous surface decontamination procedure using a mini sandblaster device with aluminium oxide powder applied under controlled pressure. This mechanical cleaning step was essential to remove potential exogenous DNA contaminants from the outer surface of the samples, thereby minimising the risk of cross-contamination and ensuring the integrity of the endogenous genetic material. Subsequently, the samples were irradiated with UV light for 30 min to ensure the complete inactivation of any potential DNA-contaminating molecules. Ancient DNA was subsequently extracted via a validated minimally non-destructive protocol [60], which allows the preservation of the physical integrity of the skeletal element throughout the entire process, enabling the bone/teeth to be returned after analysis. This approach is particularly relevant in contexts where the study of human remains is central, such as in forensic and bioarchaeological contexts, in which sample conservation is critical.

#### 2.2.2. aDNA Quantification

Following extraction, two complementary quantification strategies were employed to assess the aDNA quality and quantity, both performed at the Genomics Unit of the Research Support Centre for Biological Techniques (CAI Técnicas Biológicas) of the Complutense University of Madrid (UCM) in Spain. Total double-stranded DNA (dsDNA) was measured using the Qubit Fluorometer with the High Sensitivity (HS) assay kit (Thermo Fisher Scientific, Waltham, MA, EUA), providing a sensitive and accurate estimate of overall aDNA yield. Nuclear aDNA quantification, including the estimation of Y-chromosomal aDNA fragment presence, was subsequently performed using the Quantifiler™ Trio DNA Quantification Kit (Applied Biosystems, Thermo Fisher Scientific, Foster City, CA, EUA) following the manufacturer’s recommendations. This dual quantification approach allowed for a comprehensive evaluation of aDNA preservation and provided a preliminary indication of the biological sex of each individual through the detection of Y-chromosomal targets.

#### 2.2.3. XX and XY Genetic Profiles Estimation

To ensure high-confidence sex identification, a multiple complementary approach was implemented. Autosomal and Y-chromosomal loci were targeted during the real-time PCR quantification step, as described above. For confirmatory sex typing, extracted aDNA was amplified using the GlobalFiler™ IQC PCR Amplification Kit (Applied Biosystems, Thermo Fisher Scientific, Foster City, CA, EUA), which includes amplification of the amelogenin locus, as well as a Y-chromosomal insertion/deletion (INDEL) marker, enabling reliable differentiation between XX (female) and XY (male) genetic profiles. Additionally, Y-chromosomal Short Tandem Repeat (Y-STR) profiling was performed using the Yfiler™ Plus PCR Amplification Kit (Applied Biosystems, Thermo Fisher Scientific, Foster City, CA, EUA), further supporting male lineage estimation and enhancing the robustness of sex estimation, particularly in samples with degraded or low-template aDNA. Fragment analysis was carried out at the Genomics Unit of the Research Support Centre for Biological Techniques (Centros de Apoyo a la Investigación (CAI) Técnicas Biológicas) of the Complutense University of Madrid (UCM) on a capillary electrophoresis platform. Allelic designation was performed using GeneMapper™ ID-X Software v1.5 (Applied Biosystems, Thermo Fisher Scientific, Foster City, CA, EUA), with appropriate commercial allelic ladders and validated in-house reference panels.

#### 2.2.4. Authenticity and Interpretation Criteria

To minimise the risk of contamination and ensure the authenticity of the genetic results, a rigorous multi-layered strategy was implemented throughout the entire analytical process. For each individual, aDNA was extracted independently from two sub-samples by two different researchers on different days using distinct reagent batches. All quantification and amplification procedures were likewise performed independently and on separate days for each extraction. Between analytical sessions, laboratory surfaces, equipment, and workspaces were systematically decontaminated using UV irradiation and DNA-degrading chemical agents to eliminate any residual genetic material that could compromise subsequent analyses. Pre- and post-amplification procedures were carried out in physically separated areas to prevent cross-contamination. All work was performed under strict aseptic conditions, with personnel wearing full protective equipment, including gloves, masks, and dedicated laboratory clothing. Negative extraction and amplification controls were included in every analytical batch to monitor for environmental or reagent-derived contamination. Additionally, a reference DNA database of all laboratory personnel involved was maintained, allowing the identification and exclusion of any potential staff-derived contamination from the results.

To ensure the reliability and authenticity of the genetic results, strict interpretation criteria were applied prior to the acceptance of any allelic call. For fragment analysis only peaks exceeding the Analytical Threshold (AT) of 50 Relative Fluorescence Units (RFUs) were considered as genuine alleles; signals falling below this threshold were regarded as background noise and excluded from interpretation.

Furthermore, a result was only accepted if reproducible. Preferentially, the allelic profile was required to appear in at least one amplification from each of the two independent extractions of the same individual. When one extraction repeatedly failed to produce a result after two independent amplification attempts, the profile obtained from the successful extraction was accepted only if confirmed in a minimum of two amplifications of that same extract. These criteria were applied to both the amelogenin locus and the Y-chromosomal INDEL marker.

Regarding aDNA quantification, Quantifiler™ Trio results were considered valid exclusively when the quantified nuclear aDNA concentration exceeded 0.001 ng/µL. Given that this study involved critical, low-template DNA rather than fresh biological samples, a quantification threshold of 0.001 ng/µL was implemented. Although this value sits at the operational Limit of Detection (LOD < 1 pg/µL) of the Quantifiler™ Trio kit and carries an inherent risk of stochastic effects and allelic dropout, its application is strictly supported by the laboratory’s internal validation studies and internal Standard Operating Procedures (SOPs), which demonstrate that samples at this threshold can successfully yield forensically informative partial STR profiles. As with fragment analysis, this minimum threshold was required in both extractions of the same individual whenever possible. When one extraction repeatedly failed to produce a quantifiable result, the result from the remaining extraction was accepted only upon demonstration of reproducibility across a minimum of two independent quantification replicates.

## 3. Results

### 3.1. Bioanthropological Sex Estimation

Sex could not be estimated for more than half of the individuals (n = 19, 51.4%), including the three non-adult individuals (two children and one infant). In the remaining cases the majority were classified as “female” (n = 7, 18.9%) or “possible female” (n = 5, 13.5%). The remaining individuals were classified as “male” (n = 2, 5.4%) and “possible male” (n = 4, 10.8%) (Table 1). Skeletally mature individuals were identified by a fully developed skeleton, including the complete fusion of the sternal end of the clavicle and the vertebral ring epiphyses, which are among the last skeletal structures to undergo maturation. Consequently, all individuals assessed as having a mature skeleton were aged as being 30 years or older at time of death [6]. In the case of the juvenile individuals, age-at-death varied between five years based on the maximum length of radius, and five to ten months based on the development and eruption of the teeth [54,55,56]. The overall taphonomic preservation of the remains significantly impacted the visibility of the bones’ anatomical traits used for sex estimation. Consequently, when both the skull and os coxae were compromised, due to fragmentation for example, adult individuals were classified as “indeterminate”, thus demonstrating the limitations of morphological sex estimation and the relevance of preservation.

**Figure 1:**
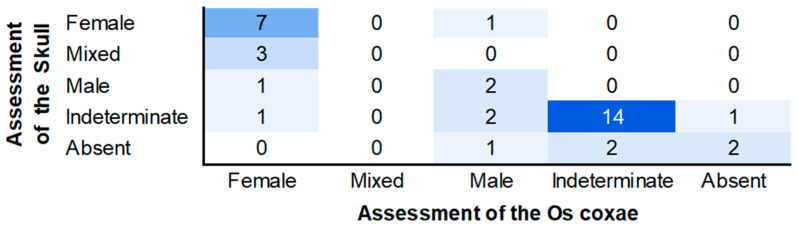
Overlap matrix of the skull (cranium and mandible) versus os coxae assessment and subsequent sex estimation.

The overlap matrix (Figure 1) shows a high degree of disagreement between the estimated sex for the skull and os coxae when sex estimation was possible. The figure outlines the macroscopic distribution of morphological traits. Among the elements that allowed for a morphological assessment, the hip-associated features yielded a substantially higher number of sex estimations, with 12 female and 6 male individuals. The skull (i.e., the cranial and mandibular features) provided more conservative estimates (females = 8, males = 3), thus introducing some ambiguity in the sex estimation of these individuals. For example, three individuals exhibited a mixture of male and female traits within the craniofacial complex. The overall consensus includes seven concordant females, two concordant males, and 14 individuals whose poor preservation rendered both sex estimations based on the skull and os coxae indeterminate (Figure 1).

### 3.2. XX and XY Genetic Profiles Estimation Results

**Figure 2:**
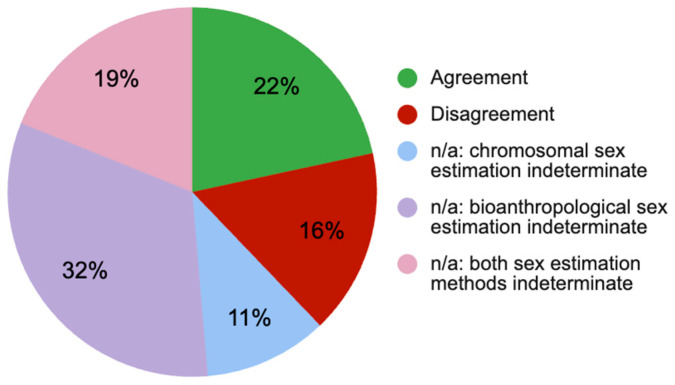
Sex estimation agreement between bioanthropological and chromosomal methods. n/a: identify percentages of cases where only one or both approaches yielded an indeterminate result.

Twenty-six individuals had a successful XX and XY genetic profiles estimation via quantitative PCR (qPCR) targeting the Y chromosome and amelogenin locus, and/or by Y-chromosome STRs. As previously indicated, genetic sex was assigned whenever the criteria established in Section 2.2.4 were met. In this sense, the estimation of chromosomal sex using genetic methods was carried out by three separate researchers, and only when there was consensus on the sex assignment, was it considered a positive result (Figure 2). To assign the sex as “male” (for example), both samples had to produce an XY result in at least two of the independent amplifications in at least one method; if one of the samples did not have a sufficient genetic concentration to obtain a result, then the other sample had to produce an XY result in at least two amplifications using at least two different methods.

In 11 cases, it was not possible to assign a sex to the individual, either due to a lack of reproducible results or the complete absence of alleles in the amelogenin gene, largely due to the low genetic concentration detected (which did not exceed 0.001 ng/µL). For this reason, it is more prudent to leave the matter open, allowing for further sampling, than to assign the female sex (due to the absence of the Y chromosome), since the absence of alleles in the amelogenin gene (both Y and X) indicates a scarcity and/or fragmentation of the information present at this locus.

### 3.3. Combined Bioanthropological and XX and XY Genetic Profile Estimation

**Figure 3:**
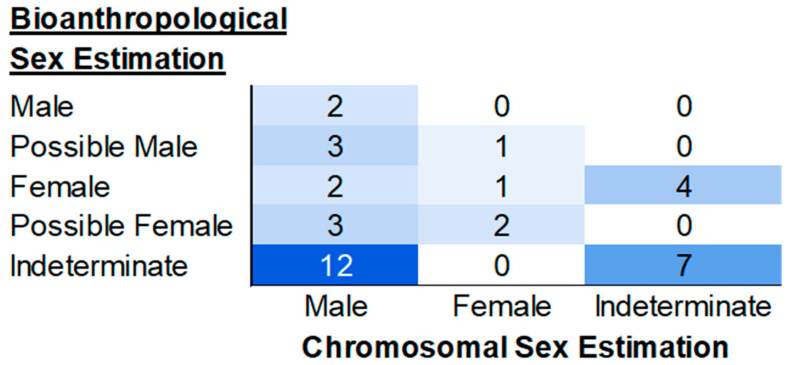
The heatmap shows the cross-tabulation of findings between the bioanthropological and chromosomal sex estimation approaches, highlighting where the two intersect, confirm each other, or diverge.

Considering the cases where sex was assigned by one or both approaches, sex estimation was possible in 30 individuals. Sex estimation agreement between bioanthropological and genetic methods was observed in 26.7% (n = 8) of cases (Figure 2). A substantial proportion of individuals initially assessed as “indeterminate” (n = 19) based on morphological traits were identified as male through aDNA analysis (n = 12/19) (Figure 3, Table 2). Where sex was estimated by both methods, 20% (n = 6) showed a direct disagreement between the physical skeleton and the genetic data. Most notably, five individuals whose remains were estimated to be “female” or “possible female”, based on the bioanthropological analysis, were found to be genetically “male” (detailed in Table 2). However, these global percentages must be interpreted with caution. If individuals with indeterminate or missing data were excluded from a direct binary comparison (n = 14), the strict inter-method discordance rate would rise to 42.9% (6/14). However, because “indeterminate” is a classification used in forensic, bioanthropological, and bioarchaeological sex estimation studies, rather than being viewed as missing data, these were included in the analysis. Retaining all 30 individuals captures the total resolving power of this multidisciplinary approach.

## 4. Discussion

The genetic and morphological results presented offer a critical appraisal of 20th-century sex estimation standards when applied to a selected sample of a 19th-century Portuguese population. The results illustrate that genetic sex estimation brings a clear contribution to the estimation of sex in cases where morphological assessment is poor or absent. Results also showed that in the sub-sample of human remains from the Carmo burial site, population-specific reduction in skeletal sexual dimorphism exists, in which males exhibit morphological traits typically associated with female estimation, i.e., more gracile features, leading to the systematic misclassification under standard osteological criteria. These findings differ from the Portuguese literature, as studies carried out in identified reference collections have reported male accuracy rates between 70 and 80% for the skull and 90% and higher for the os coxae [13,17,61]. It is possible that these findings were not replicated in this study, due to the studies having much larger sample sizes, diverging levels of sexual dimorphism, and/or the existence of socio-temporal differences between the Carmo sub-sample and the identified reference collections (although these comprise a significant overlap in chronology). The obtained results underscore the limitations of applying universal standards based on morphological traits for sex estimation in the Carmo sample. However, rather than suggesting a structural shift toward more delicate skeletal morphological traits among 19th-century males from Porto, these results indicate that the observed trend reflects inherent population variability. The tendency of the sub-sample to express traits traditionally categorised as female under standard forensic/anthropological methods highlights how such classifications are tied to historical descriptive narratives rather than absolute morphological boundaries.

Also, it emphasises the fact that bone tissue is highly plastic and sensitive to several factors, including nutritional stress, health status, and occupational activities, shaping a population’s sexual dimorphism. Consequently, one cannot blindly apply sex estimation methods without considering confounding variables, not forgetting age-at-death. Additionally, in the context of the Carmo sub-sample associated with a hospital cemetery, socio-economic and biological pressures would have impacted the skeleton. For example, individuals who were bedridden due to illness(es) would have faced decreased biomechanical loading, which would have likely affected their overall skeletal morphology.

The resolution of most indeterminate cases through genetic analysis supports the value of genetic validation as an important complementary method to bioanthropological sex estimation for accurate demographic profiling. This is especially true for fragmented remains as well as non-adult remains in which sexual dimorphism is incipient or, as in this case, where it is related minimally to chromosomal sex. Genetic analysis represents, arguably, the most reliable method currently available for sex estimation in skeletal and dental remains. Unlike morphological or morphometric approaches, genetic sex estimation is entirely independent of interindividual variation in physical development, nutritional status, mechanical loading history, pathological conditions, or sociocultural and temporal context. Chromosomal sex is encoded in every nucleated cell of an organism. Furthermore, genetic sex estimation is age-independent: the chromosomal information is identical in a foetus, a neonate, a young adult, or an elderly individual, rendering skeletal maturation or the developmental stage entirely irrelevant to the analysis. Equally, genetic methods are sample-type universal: the same analytical approach is applicable to bone, dentine, blood, or saliva, with no requirement for population-specific reference data, allowing for a direct and explicit estimation of chromosomal sex regardless of the archaeological, forensic, or clinical context.

It is, however, critically important to distinguish chromosomal information from “*gender*”, which reflects an individual’s social construct, role, identity, and cultural expression [62,63,64,65]. Genetic analysis identifies the chromosomal information of an individual—XX, XY, XXY, XYY, among other configurations—and provides no information whatsoever regarding how that individual identified socially, what roles they occupied within their community, or their personal sense of identity. Furthermore, depending on context, “sex” may variably refer to chromosomal status, hormonal profile, or expression of various anatomical structures—all of which exhibit their own spectrum of expressions, which in turn may co-occur in various combinations [65]. Conflating sex with gender constitutes a fundamental conceptual error: these are distinct but interrelated constructs, and the results of genetic sex estimation constitute a crucial component of multifactorial sex estimation, which allows for holistic interpretation of the sociosexual lives of past people [65,66,67,68].

As highlighted in this paper, the principal limitation of genetic sex estimation lies in (a)DNA preservation, not excluding issues related with cost and logistics (the need for equipment, laboratory and specialised staff). When nuclear genetic material is severely degraded, amplification of sex-linked loci may fail entirely or yield incomplete profiles. Critically, the detection of a single X-chromosomal signal must never be interpreted as indicative of a female (XX) genotype. The absence of a Y-chromosomal signal in a highly degraded sample may reflect allelic dropout rather than a true XY-negative constitution, meaning that the individual could be XX, XY, or other configurations. Accordingly, genetic profiles exhibiting a high proportion of allelic dropout or locus failure must be interpreted with the utmost caution, and any sex estimation based on such profiles should be explicitly flagged as inconclusive. The integration of genetic data to refine population-specific morphological criteria and enhance the accuracy of biological profiling, particularly in contexts where factors such as age, the environment, and nutrition may influence skeletal morphology, is crucial. Morphological sex estimation has limitations with poor bone preservation, particularly high levels of fragmentation, poor surface preservation, and incomplete remains. Owing to local geochemical conditions, such as acidic and waterlogged soil, all of these limitations affect the remains investigated here to some extent.

### Ethical Considerations on Genetic Sex Estimation

As with all ancient DNA research, the application of genetic methods to archaeological skeletal remains can raise ethical considerations that must be acknowledged [69]. These include the partially destructive nature of sampling, even when minimised through validated non-destructive protocols, and the impossibility of obtaining informed consent from the deceased, relatives, and/or associated communities. Also, cultural and community sensitivities are of particular relevance when the individuals under study belong to native or indigenous populations, vulnerable communities, or to specific groups whose known living descendants have not been consulted prior to the research. In such cases, engagement with descendant communities should be considered a fundamental ethical requirement [70,71,72,73].

The ethical concerns regarding sex estimation in ancient DNA research represent a necessary evolution in our responsibility. Sex estimation of archaeological human remains, whether through osteological, genetic or other means, requires a number of specific ethical considerations in their interpretations and reporting. Sex is a complex socially constructed concept constituted by many component parts such as chromosomes, gross anatomy, hormones, and more [65,66,67,68]. Nonetheless, sex is still commonly reported as simply male and female (or indeterminate if in doubt). This binary model of sex flattens the diversity and complexity of human biology and runs the risk of being used to underpin overly simplistic narratives of the gendered lives of archaeological populations [45,65,66,67]. This model also frequently reduces sex, as well as individuals themselves, to singular component parts, such as specific body parts, or chromosomal makeup [68]. With the advancement of biomolecular techniques, a molecularisation of identity has emerged, which frequently falls back into deterministic understandings of sex and gender [45,68]. Thus, care must be taken when interpreting biomolecular sex estimations to not reproduce “biology as destiny”, particularly so if the biology in question constitutes just one of the many components of sex. Reenforcement of binary notions of sex also render intersex persons invisible in the archaeological record, as there are currently no routinely applied genetic methods to detect such individuals [65].

Additional ethical issues relate to the absence of consent to access aDNA, including from descent communities and/or associated communities, which ties to issues related to privacy. Notably, ethical issues related to privacy issues of potential living relatives of ancient/historical individuals warrant rigorous scientific scrutiny before being viewed as a genuine ethical issue in archaeological contexts. This is because genetic identification, whether of a living or deceased individual, relies on a direct comparison between an unknown profile and a known reference sample. In the case of archaeological human remains, assigning an individual identity would therefore require either a biological sample from the person concerned or from demonstrably related individuals. This applies to someone from the 21st century, the 19th century, or the 1st century BCE. In many archaeological cases, known living descendants cannot be identified with confidence, and there is no comprehensive reference database of deceased individuals or their relatives. As a result, even a well-preserved or complete genetic profile from an ancient individual usually cannot be linked to a named living person. In some circumstances analysis may be extended to potential relatives, but this typically addresses biological relatedness rather than the identification of the individual themselves. Moreover, shared mitochondrial or Y-chromosomal haplotypes between ancient and living individuals should be interpreted cautiously, as these often reflect broad lineage or population-level ancestry rather than direct kinship [66,67]. Since concepts of kinship and family are also culturally defined, privacy concerns are most directly relevant where living individuals, or communities, have a relationship of descent or affiliation with the remains. This is exemplified in the identification of 20th-century victims of state violence and armed conflicts, such as the Spanish Civil War, South American dictatorships, the Bosnian War, or the World Wars, as well as descent communities [70,71,72,73,74,75,76].

On a final note, genetic sex estimation is admittedly more resource-intensive than bioanthropological approaches. However, this investment is justified by the objectivity, reproducibility, and independence from taphonomic and developmental variables that characterise genetic methods, rendering them uniquely reliable in contexts where skeletal preservation is poor or morphological indicators are ambiguous. Ethical issues also extend to what happens to the data after they are generated. Hence, genetic sex estimation is not always just about “sex” and “biology”.

## 5. Conclusions

The comparison between macroscopic and genetic sex estimation in the Carmo sub-sample reveals that traditional osteological methods can be misleading. The identified 20% (n = 6/30) discordance rate serves as a stark reminder that human skeletal variation is highly plastic and responsive to the environment and behaviour. While morphology remains a valuable and accessible tool, it must be used with a critical awareness of population-specific dimorphism, and the importance of considering environmental and historical data when assessing a population. Genetic analysis, particularly using non-destructive methodological approaches, may be a way of resolving ambiguous cases. However, care is also necessary in its use and interpretation of the results. Genetic sex estimation is a powerful tool precisely because of its reproducibility and independence from taphonomic issues, but its interpretive authority must be tempered by an awareness that chromosomal information is but one aspect within a far richer tapestry of biological and social representation of people. Genetic sex estimation offers a degree of resolution that bioanthropological methods alone cannot match, yet its strength lies as much in how its results are situated as in how they are produced.

## Figures and Tables

**Table 1:** Bioanthropological sex estimation summary.

Field Identification *	Bone Maturation Stage	Skull	Os Coxae	Sex Estimation
PCM22_UE2596	Mature skeleton	Damaged	Damaged	Indeterminate
PCM22_UE2616	Mature skeleton	Damaged	Damaged	Indeterminate
PCM22_UE2627	Mature skeleton	Absent	Absent	Indeterminate
PCM22_UE2628	Mature skeleton	Damaged	Damaged	Indeterminate
PCM22_UE2636	Mature skeleton	Damaged	Damaged	Indeterminate
PCM22_UE2638	Mature skeleton	Damaged	Damaged	Indeterminate
PCM22_UE2639	Mature skeleton	Damaged	Absent	Indeterminate
PCM22_UE2730	Mature skeleton	Damaged	Damaged	Indeterminate
PCM22_UE2827	Mature skeleton	Absent	Damaged	Indeterminate
PCM22_UE2828	Mature skeleton	Damaged	Damaged	Indeterminate
PCM22_UE2866	Mature skeleton	Damaged	Damaged	Indeterminate
CAL32.06_UE941	Mature skeleton	Absent	Indeterminate	Indeterminate
CAL32.06_UE943	Mature skeleton	Female features (both)	Female features	Female
CAL32.06_UE5072	Mature skeleton	Damaged	Damaged	Indeterminate
CAL32.06_UE5267	Mature skeleton	Female features (both)	Female features	Female
CAL32.06_UE5335	Mature skeleton	Female features (both)	Female features	Female
CAL32.06_UE5336	Mature skeleton	Mixture of male and female features (both)	Female features	Possible female
CAL32.06_UE5908	Mature skeleton	Female features (both)	Female features	Female
CAL32.06_UE5949	Mature skeleton	Male features (both)	Male features	Male
CAL32.06_UE5989	Mature skeleton	Female features (cranium)	Female features	Female
CAL32.06_UE6062	Mature skeleton	Absent	Absent	Indeterminate
CAL32.06_UE6126	Mature skeleton	Female features (both)	Female features	Female
CAL32.06_UE6128	Mature skeleton	Damaged	Damaged	Indeterminate
CAL32.06_UE6186	Mature skeleton	Absent	Male features	Possible male
CAL32.06_UE6190	Mature skeleton	Mixture of male (mandible) and female features (cranium)	Female features	Possible female
CAL32.06_UE6201	Mature skeleton	Indeterminate	Indeterminate	Indeterminate
CAL32.06_UE6348	Mature skeleton	Damaged	Female features	Possible female
CAL32.06_UE6363	Mature skeleton	Mixture of male (mandible) and female features (cranium)	Female features	Possible female
CAL32.06_UE6364	Mature skeleton	Female features (both)	Female features	Female
CAL32.06_UE6394	Mature skeleton	Female features (both)	Male features	Possible male
CAL32.06_UE6410	Mature skeleton	Male features (cranium)	Female features	Possible female
CAL32.06_UE6433	Mature skeleton	Male features (cranium)	Male features	Male
CAL32.06_UE6434	Mature skeleton	Damaged	Male features	Possible male
CAL32.06_UE6435	Mature skeleton	Damaged	Male features	Possible male
PCM22_UE2595	Immature skeleton ** (child ~ 5 years old)	Indeterminate	Indeterminate	Indeterminate
PCM22_UE2850	Immature skeleton ** (infant between 5 and 10 months)	Indeterminate	Indeterminate	Indeterminate
CAL32.06_UE5948	Immature skeleton ** (child ~ 2 years old)	Indeterminate	Indeterminate	Indeterminate

* Field identification includes the acronym and year of the project—PCM22 (Palácio do Carmo 2022) or CAL32.06 (Carlos Alberto Square, 32 police number, year 2006) and UE (stratigraphic unit or context number). ** CAL32.06_UE5948 non-adult individual of around 2 years +/− 8 months of age; estimation is based on the development and eruption of the teeth. PCM22_UE2595 non-adult individual of around 5 years of age based on the maximum length of radius. PCM22_UE2850 non-adult individual between 5 and 10 months of age based on dental development and eruption.

**Table 2:** Summary of bioanthropological and genetic sex estimation results for each individual, with Agreement and Disagreement indication.

Field Identification *	Bioanthropological Sex Estimation	Chromosomal Sex Estimation	Agreement/Disagreement Between Bioanthropological and Genetic Sex Estimation
CAL32.06_UE6394	Possible male	Female	Disagreement
CAL32.06_UE5336	Possible female	Male	Disagreement
CAL32.06_UE6348	Possible female	Male	Disagreement
CAL32.06_UE6410	Possible female	Male	Disagreement
CAL32.06_UE5908	Female	Male	Disagreement
CAL32.06_UE6126	Female	Male	Disagreement
CAL32.06_UE941	Indeterminate	Male	N/a: bioanthropological sex estimation indeterminate
CAL32.06_UE6062	Indeterminate	Male	N/a: bioanthropological sex estimation indeterminate
CAL32.06_UE6128	Indeterminate	Male	N/a: bioanthropological sex estimation indeterminate
PCM22_UE2627	Indeterminate	Male	N/a: bioanthropological sex estimation indeterminate
PCM22_UE2636	Indeterminate	Male	N/a: bioanthropological sex estimation indeterminate
PCM22_UE2638	Indeterminate	Male	N/a: bioanthropological sex estimation indeterminate
PCM22_UE2730	Indeterminate	Male	N/a: bioanthropological sex estimation indeterminate
PCM22_UE2827	Indeterminate	Male	N/a: bioanthropological sex estimation indeterminate
PCM22_UE2866	Indeterminate	Male	N/a: bioanthropological sex estimation indeterminate
CAL32.06_UE5948 **	Indeterminate	Male	N/a: bioanthropological sex estimation indeterminate
PCM22_UE2850 **	Indeterminate	Male	N/a: bioanthropological sex estimation indeterminate
PCM22_UE2595 **	Indeterminate	Male	N/a: bioanthropological sex estimation indeterminate
CAL32.06_UE943	Female	Indeterminate	N/a: chromosomal sex estimation indeterminate
CAL32.06_UE5267	Female	Indeterminate	N/a: chromosomal sex estimation indeterminate
CAL32.06_UE5335	Female	Indeterminate	N/a: chromosomal sex estimation indeterminate
CAL32.06_UE5989	Female	Indeterminate	N/a: chromosomal sex estimation indeterminate
CAL32.06_UE5949	Male	Male	Agreement
CAL32.06_UE6364	Female	Female	Agreement
CAL32.06_UE6433	Male	Male	Agreement
CAL32.06_UE6186	Possible male	Male	Agreement
CAL32.06_UE6190	Possible female	Female	Agreement
CAL32.06_UE6363	Possible female	Female	Agreement
CAL32.06_UE6434	Possible male	Male	Agreement
CAL32.06_UE6435	Possible male	Male	Agreement
CAL32.06_UE5072	Indeterminate	Indeterminate	N/a: both bioanthropological and chromosomal sex estimation indeterminate
CAL32.06_UE6201	Indeterminate	Indeterminate	N/a: both bioanthropological and chromosomal sex estimation indeterminate
PCM22_UE2596	Indeterminate	Indeterminate	N/a: both bioanthropological and chromosomal sex estimation indeterminate
PCM22_UE2616	Indeterminate	Indeterminate	N/a: both bioanthropological and chromosomal sex estimation indeterminate
PCM22_UE2628	Indeterminate	Indeterminate	N/a: both bioanthropological and chromosomal sex estimation indeterminate
PCM22_UE2639	Indeterminate	Indeterminate	N/a: both bioanthropological and chromosomal sex estimation indeterminate
PCM22_UE2828	Indeterminate	Indeterminate	N/a: both bioanthropological and chromosomal sex estimation indeterminate

* Field identification includes the acronym and year of the project—PCM22 (Palácio do Carmo, year 2022) or CAL32.06 (Carlos Alberto Square, 32 police number, year 2006) and UE (stratigraphic unit or context number). ** Identifies the non-adult cases. N/a: One or both methods did not result in either female or male sex classifications, due to bone damage, bone absence, or an inconclusive result. Chromosomal sex was estimated through a multi-marker approach, including the detection of Y-chromosomal aDNA by the Quantifiler™ Trio DNA Quantification Kit, amelogenin locus typing, and Y-chromosomal INDEL marker analysis, the latter two performed using the GlobalFiler™ IQC PCR Amplification Kit (Thermo Fisher Scientific).

## Data Availability

Data supporting the results will be made available in the BeFRAIL Zenodo community (https://zenodo.org/communities/befrail/records?q=&l=list&p=1&s=10&sort=newest). All bioanthropological data assessment will be made available upon conclusion of the ongoing research (estimated date December 2028). However, even after that period, some bioanthropological data may have an embargo associated to it, due to sensitivity reasons, and to comply with the BeFRAIL ethical assessment protocol. The raw aDNA data will not be openly archived, but will be made available upon reasonable request.
